# Bcl-2 regulates pyroptosis and necroptosis by targeting BH3-like domains in GSDMD and MLKL

**DOI:** 10.1038/s41420-019-0230-2

**Published:** 2019-12-09

**Authors:** Chong-Shan Shi, John H. Kehrl

**Affiliations:** B Cell Molecular Immunology Section, Laboratory of Immunoregulation, National Institute of Allergy and Infectious Diseases, National Institutes of Health, Bethesda, MD 20892 USA

**Keywords:** Necroptosis, Immune cell death

## Abstract

Apoptosis is a form of programmed cell death in multicellular organisms. Bcl-2 prevents apoptosis and promotes cellular survival by neutralizing BH3 domain-containing proteins, which directly activate the pore-forming proteins BAX and BAK. However, Bcl-2 is not known to regulate other cell death effectors such as gasdermin D (GSDMD) or mixed lineage kinase domain-like (MLKL), whose activation causes pyroptosis and necroptosis, respectively. Here, we identify a BH3-like domain in both GSDMD and MLKL that mediates an interaction with B-cell lymphoma 2 (Bcl-2). The presence of Bcl-2 reduced GSDMD cleavage at D275 by caspase-1, 4 or 5, and enhanced the GSDMD cleavage at D87. The GSDMD D87 cleavage inactivates the pyroptotic execution program. The presence of Bcl-2 also limited RIP3 mediated phosphorylation of MLKL, which reduced MLKL oligomerization and tempered the induction of necroptosis. Our observations suggest that the presence of Bcl-2 limits the induction of three forms of cell death apoptosis, pyroptosis, and necroptosis.

## Introduction

Apoptosis helps preserve tissue homeostasis and is obligatory for normal embryonic development^1^. The downregulation of apoptosis can contribute to tumorigenesis, while its upregulation functionally impacts the severity of a variety of infectious, neurodegenerative, and autoimmune diseases^2^. The intrinsic pathway also known as the mitochondrial apoptotic pathway is tightly regulated by members of the Bcl-2 protein family. Together these protein function to modulate the permeability of the mitochondrial outer membrane^3^.

Based on their operational roles in apoptosis and the number of Bcl-2 homology (BH) domains that they possess, the Bcl-2 family members are divided into three subgroups^4^. One subgroup includes the proapoptotic members of the family such as Bax and Bak. These proteins mediate the increased mitochondrial outer membrane permeability. A second subgroup are the BH3-only members Bid, Bim, and PUMA, which are the initiators of the apoptosis program. The third subgroup includes the antiapoptotic or pro-survival Bcl-2 family members such as BCL-2, BCL-XL, BCL-W, and MCL1. Each of the pro-survival proteins possess four BCL-2 homology domains (BH1-4), which form a furrow-like structure in the individual proteins. Lacking a cell death signal, the furrow in the pro-survival proteins contacts the pro-apoptotic BH3 proteins, thereby blocking their activation. Moreover, the pro-survival proteins also bind the pro-apoptotic effectors BAX and BAK, which limits their oligomerization. A variety of non-receptor mediated events can trigger the intrinsic apoptosis pathway including growth factor removal, radiation, and certain toxins. The BH3-domain only proteins initiate the apoptosis program by releasing BAX or BAK from the pro-survival BCL-2-like proteins. They also directly bind and activate BAX and/or BAK^5,6^. The activated BAX and BAK proteins auto-oligomerize forming pore-like structures in the mitochondrial outer membrane causing the release into the cytosol of pro-apoptotic mitochondrial contents. Among them is cytochrome c, which facilitates the oligomerization of APAF-1 (apoptotic peptide activating factor-1). Forming an heptameric structure, APAF-1 recruits and activates caspase-9, thereby forming the apoptosome, which cleaves and activates the effector caspases^7^.

Using genomic screening techniques, two groups in 2015 reported the identification of GSDMD as a substrate of inflammasome-associated caspase-1, 4 or 5 in humans and caspase-1 and 11 in mice. These caspases cleave GSDMD to generate N-terminal fragments that translocate to cellular membranes, auto-oligomerize, and assemble into membrane pores. This facilitates the secretion of IL-1β and IL-18; and triggers an inflammatory programmed cell death process called pyroptosis^8,9^. Functionally, pyroptosis is a major mediator of lethal polymicrobial sepsis^10^. In neutrophils, GSDMD cleavage helps generate neutrophil extracellular traps, a specialized form of neutrophil cell death that releases chromatin and antimicrobial proteins into the extracellular milieu^11,12^. Active caspase-1 cleaves and triggers caspase-3 and caspase-7 activation through an unclear mechanism, which in turn cleave GSDMD at position D87 in the protein. This inactivates GSDMD pyroptosis in monocytes and macrophages^13^.

Necroptosis is a form of regulated necrotic cell death that is mediated by the RIP1/RIP3/MLKL signaling axis. Engagement of cell death receptors activates RIP1 and RIP3. The activated RIP kinases form a large protein complex called a necrosome, which phosphorylates MLKL^14–16^. The phosphorylated MLKL proteins translocate to the plasma membrane, where together with the inositol phosphate (IP) kinases IPMK and ITPK1 they form a highly phosphorylated protein complex, which facilitates the displacement of the MLKL auto-inhibitory brace region. This promotes MLKL auto-oligomerization and membrane pore formation to initiate necroptosis^17^.

The prominent functional role of Bcl-2 proteins in protecting cells from apoptosis prompted us to assess whether Bcl2 proteins might affect other forms of cell death. Here, we show that GSDMD and MLKL contain a BH3-like domain, which serves as a key structural motif to enable an interaction with Bcl-2. We find that Bcl-2 functions not only as an antiapoptotic protein, but also as an anti-pyroptotic and an anti-necroptotic protein.

## Results

### GSDMD interacts with Bcl-2

**Fig. 1:**
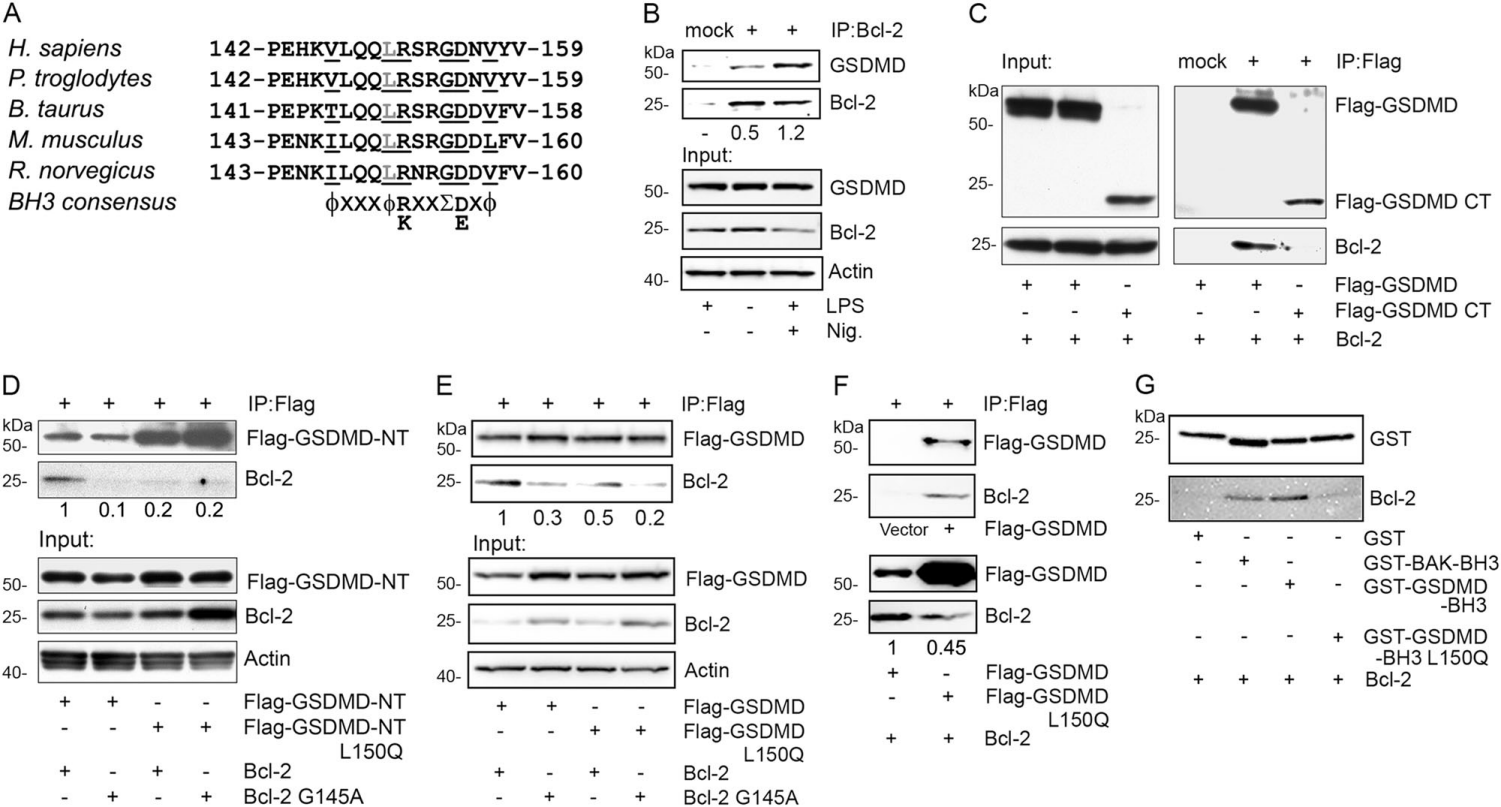
Bcl-2 interacts with GSDMD. **a** A predicted BH3-like domain in GSDMD is conserved across the indicated species. The consensus BH3 domain sequence is shown. **b** Endogenous Bcl-2 interacts with the endogenous GSDMD. Lysates from THP-1 cells treated with the indicated inflammasome activators (LPS 50 ng/ml 5 h, nigericin 5 µM 30 min) were used for Bcl-2 immunoprecipitations. The blots of Bcl-2 that co-immunoprecipitated with GSDMD are showed on the upper panel. The lower panels show the input proteins in cell lysates. **c** GSDMD-CT does not interact with Bcl-2. Lysates from HEK-293T cells transfected with the indicated constructs were immunoprecipitated using anti-Flag antibodies conjugated agarose beads to pull down Flag-GSDMD. Matched non-specific antibodies were used for the mock immunoprecipitation. The left panel shows the input proteins, and the right panel shows the interacting proteins. **d** GSDMD-NT and its BH3-like domain are required for the interaction between GSDMD and Bcl-2. The lysates from HEK-293T cells transfected with the indicated constructs were immunoprecipitated using anti-flag agarose beads. The upper panel shows the interacted proteins, and the lower panel shows the input proteins. **e** BH3-like domain of GSDMD is required for the interaction with Bcl-2. The lysates from HEK-293T cells transiently expressing the indicated constructs were used to immunoprecipitated Flag tagged GSDMD. The upper panel shows the co-immunoprecipitated proteins, and the lower panel shows the input proteins. **f** Bcl-2 recombinant protein binds GSDMD in vitro. Immunoprecipitated GSDMD or its L150Q mutant from HEK-293T cells were incubated with Bcl-2 recombinant protein, or the same amount of BSA as a negative control for one hour, then heavily washed. The upper panel shows Bcl-2 bound with GSDMD. The lower panel shows Bcl-2 bound to GSDMD wild type and L150Q mutant. **g** GST fusion BH3-like domain of GSDMD binds with Bcl-2 recombinant protein. The indicated GST fusion BH3 domains were expressed and purified from HEK-293T cells. Bcl-2 recombinant protein was added for in vitro binding with indicated GST fusion proteins.

Bcl-2 functions by binding the BH3 domain in target proteins^6^. To address whether Bcl-2 might have a similar role in the regulation of pyroptosis, we carefully examined the amino acid sequence of pyroptotic executor protein GSDMD for the presence of a BH3-like domain. The consensus sequence for a BH3 domain is **Hy-xxx-Hy-K/R-xx-Small-D/E-x-Hy**, where Hy is a hydrophobic residue and Small indicates a small amino acid such as glycine^18^. A highly conserved region in the N-terminal portion of GSDMD conforms to this consensus sequence for a BH3 domain (Fig. 1a). To verify that this region might confer upon GSDMD the ability to interact with Bcl-2, we checked whether the endogenous proteins co-immunoprecipitated using lysates prepared from the human macrophage cell line THP-1. We found that under basal conditions a constitutive interaction between Bcl-2 and GSDMD could be detected (Fig. 1b). To assess whether NLRP3 inflammasome activation and the induction of pyroptosis might affect this interaction, we primed THP-1 cells with LPS and stimulated them with nigericin. Following this treatment, the level of GSDMD in cell lysates remained similar, while that of Bcl-2 declined slightly. However, 2.4-fold more GSDMD associated with Bcl-2 than had under basal conditions (Fig. 1b).

Caspase-1, 4 or 5 cleaves human GSDMD at position 275, forming an N-terminal fragment (NT) and C-terminal fragment (CT) fragment. Consistent with the location of BH3-like domain in NT fragment of GSDMD, the CT fragment of GSDMD did not co-immunoprecipitate with Bcl-2 (Fig. 1c). Three hydrophobic residues in the BH3 domain are critical for binding BH3 domain-containing proteins within the groove of Bcl-2. When we replaced leucine 150 in the BH3-like domain of GSDMD NT with glutamine, it disrupted the interaction with Bcl-2. Furthermore, when we compared Bcl-2 and Bcl-2 G145A, which no longer binds BH3 domain proteins, we found that Bcl-2 G145A did not interact with GSDMD (Fig. 1d) When we replaced GSDMD NT fragment with its full-length version and performed the same assay, we found comparable results (Fig. 1e). To further examine whether GSDMD directly binds Bcl-2, a Bcl-2 recombinant protein was incubated with the immunoprecipitated and washed GSDMD wild type or Bcl-2 target deficient mutant L150Q. We found that Bcl-2 bound GSDMD, and that GSDMD L150Q mutation reduced its interaction with Bcl-2 (Fig. 1f). To confirm that the BH3-like domain of GSDMD alone can bind the Bcl-2 recombinant protein, we used a GST fusion of GSDMD (amino acids 142–159), or its L150Q mutant. As a positive control, we used the BAK BH3 domain (amino acids 70–87). Our data showed that the BH3-like domain of GSDMD pulled down Bcl-2 as efficiently as did the BAK BH3 domain (Fig. 1g). Taken together, the interaction between GSDMD and Bcl-2 depends on a BH3-like domain present in the N-terminal portion of GSDMD.

### Bcl-2 inhibits GSDMD cleavage by caspases 1,4, and 5

**Fig. 2:**
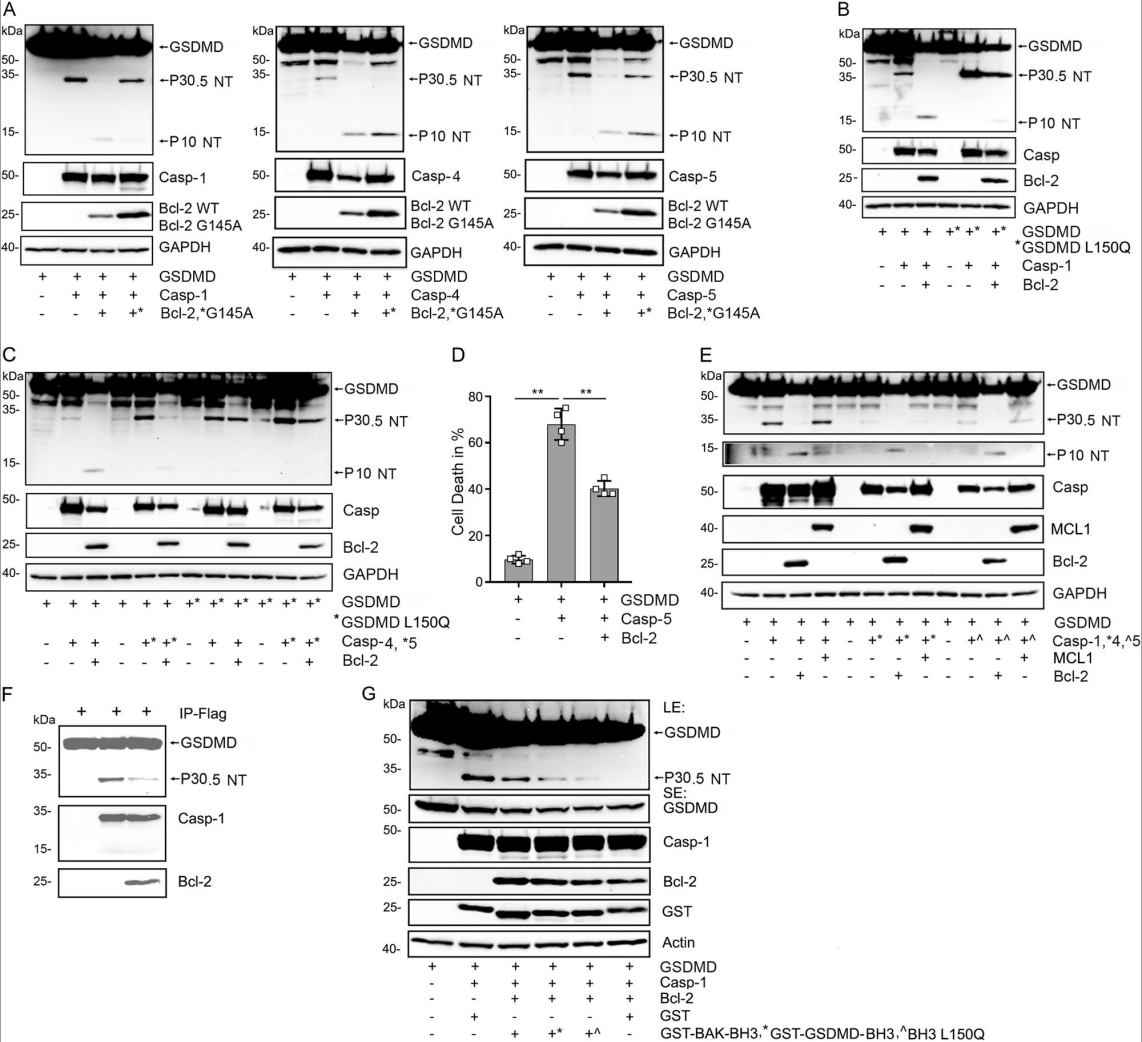
Bcl-2 reduces the caspase-1/-4/-5 generated P30.5 GSDMD fragment but enhances the P10 N-terminal fragment. **a** Cell lysates from HEK-293T cells transiently expressing the indicated constructs were used for the immunoblots. The Flag-GSDMD-full length, Flag-N-terminal (NT) fragment P30.5, and the Flag-NT fragment P10 proteins were detected with the anti-Flag antibody (upper panels), and the caspase proteins blotted with anti-Myc antibody (middle panels). The expressed Bcl-2 was immunoblotted with anti-Bcl-2 antibody. The GAPDH blot serves as a loading control. **b**, **c** BH3-like domain of GSDMD is required for Bcl-2 to regulate GSDMD cleaved by caspase-1, -4 or -5. Immunoblots of cell lysates from HEK-293T cells transiently transfected with Flag-GSDMD or Flag-GSDMD L150Q and the indicated constructs. The full length, P30.5, and P10 N-terminal fragments were detected with anti-Flag antibody (upper panels). Caspase-1/-4/-5 were detected with anti-Myc antibody. Bcl-2 was immunoblotted with anti-Bcl-2 antibody. **d** Trypan blue exclusion was used to quantitate cell death. The HEK-293T cells were transfected with the indicated constructs for 24 h. Data are means and ± SD of four samples. **p < 0.01. **e** Bcl-2, but not MCL1 regulates caspase-1, -4 or -5 cleavage of Flag-GSDMD into NT fragment P30.5 and NT fragment P10. The lysates from HEK293T cells transfected with indicated constructs and immunoblotted as above. MCL1 expression was detected with specific antibodies. **f** Bcl-2 recombinant protein inhibits the cleavage of GSDMD driven by active caspase-1 recombinant protein in vitro. The immunoprecipitated Flag-GSDMD, active form of caspase-1, and Bcl-2 protein were used for in vitro caspase-1 cleavage assay. The immunoblots show the indicated proteins. **g** GST fusion BH3-like domain of GSDMD reduces the inhibition by Bcl-2 of caspase-1 mediated cleavage of GSDMD. The indicated proteins were expressed in HEK-293T cells, the lysates were used for immunoblots. The first panel shows a long exposure (LE) of GSDMD and its P30.5 N-terminal. The second panel shows a shorter exposure (SE) of full-length GSDMD. Also shown are the indicated expressed proteins and actin levels.

Next, we expressed caspase-1, 4, or 5 in HEK 293 T to examine the GSDMD cleave pattern generated when we co-expressed Bcl-2 or its G145A mutant. We found that Bcl-2 reduced the GSDMD P30.5 NT cleavage fragment following caspase-1, 4 or 5 expression, but that the Bcl-2 mutant was less able to suppress this cleavage. Interestingly, expression of both the wild type and the mutant Bcl-2 caused the appearance of a P10 NT band (Fig. 2a). To address whether BH3-like domain of GSDMD is critical for Bcl-2 to regulate the caspase mediated GSDMD cleavage, we compared the wild type protein to the GSDMD L150Q protein. Caspase-1, 4, and 5 cleaved the GSDMD L150Q protein to form a P30.5 NT fragment as observed with the wild type GSDMD, however co-expression of Bcl-2 did not have meaningfully impact on the cleavage. The GSDMD L150Q mutation suppressed the appearance of the P10 NT fragment observed following Bcl-2 expression with the wild type GSDMD (Fig. 2b, c). Importantly, the expression of Bcl-2 reduced the amount of cell death detected following expression of caspase-5 and wild type GSDMD (Fig. 2d). To determine whether another pro-survival Bcl-2 protein family member had the same effect, we co-expressed MCL1 with either caspase-1, 4, or 5 in HEK293T cells. In contrast to Bcl-2, MCL1 did not reduce the appearance of the P30.5 NT fragments of GSDMD cleaved by the different caspases. MCL1 expression did induce the appearance of the P10 NT fragment of GSDMD when co-expressed with caspase-1, but we did not detect the P10 NT fragments following caspase-4 or caspase-5 expression (Fig. 2e). To check whether Bcl-2 recombinant protein could impair GSDMD cleavage by caspase-1 in vitro, we used immunoprecipitated GSDMD as a substrate for a recombinant active form of caspase-1. We found that Bcl-2 protein partially reduced the cleavage (Fig. 2f). Next, we assessed whether the addition of the GST-GSDMD BH3-like domain, the L150Q mutation, or GST-BAK BH3 domain would affect the Bcl-2 mediated protection. We co-expressed the various proteins in HEK293T cells. While less effective than the GST-BAK BH3 domain, the GST-GSDMD BH3-like domain partially reversed the Bcl-2 protection and was superior to the L150Q mutant protein (Fig. 2g). These data indicate Bcl-2 targets that GSDMD BH3-like domain to limit caspase cleavage.

### Bcl-2 induces cleavage of GSDMD at D87

**Fig. 3:**
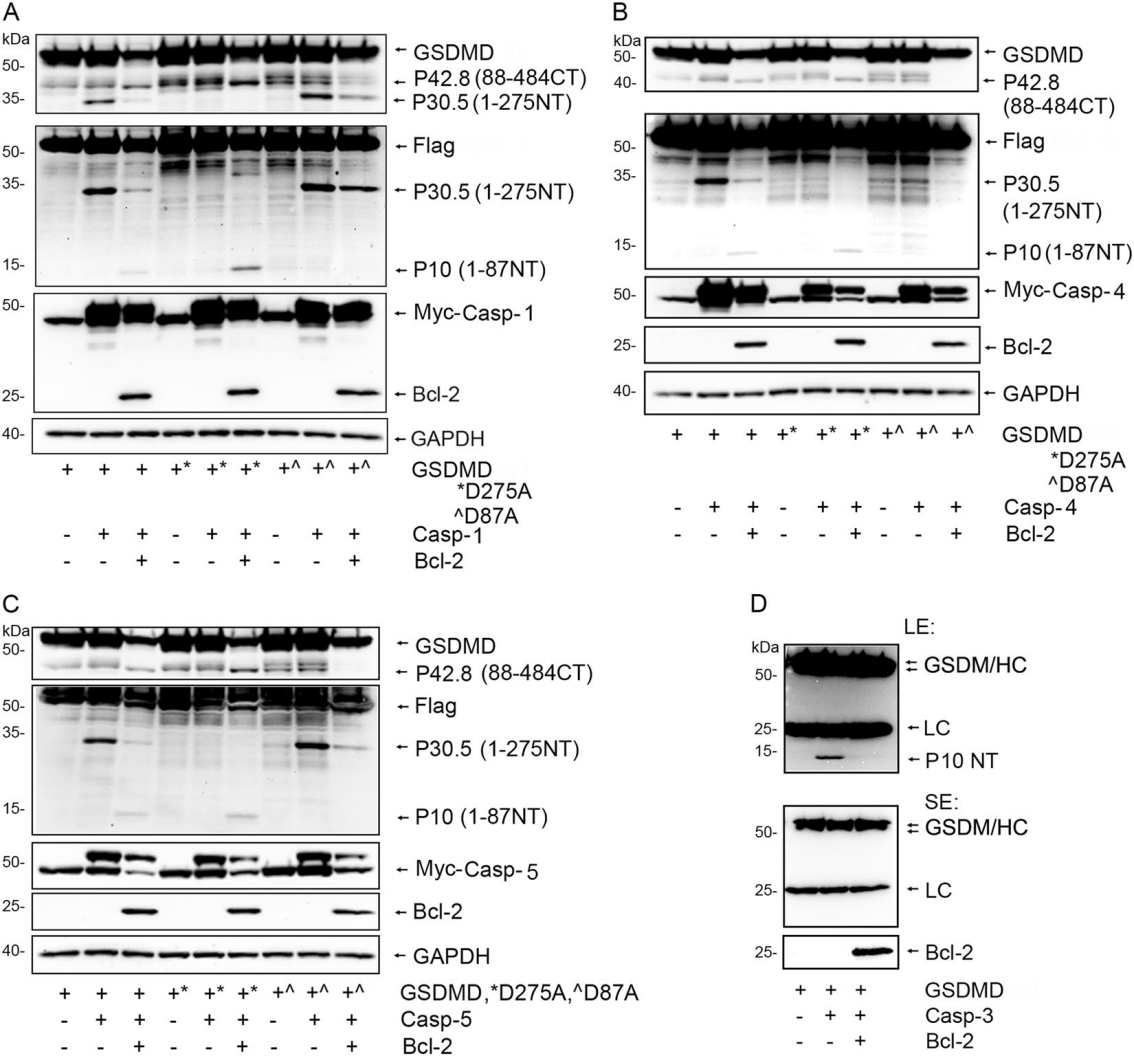
Bcl-2 induces caspase-1/-4/-5 to cleave GSDMD at D87. **a**–**c** Cell lysates from HEK-293T cells transfected with the Flag-GSDMD, Flag-GSDMD D87A, and Flag-GSDMD D275A along with Myc-Casp-1 (**a**), Myc-Casp-4 (**b**), or Myc-Casp-5 (**c**) in the presence or absence of Bcl-2 were used for immunoblotting. Antibodies raised against amino acids 154–252 of GSDMD were used to detect the C-terminal fragment P42.8 of Flag-GSDMD. The membranes were re-blotted with anti-Flag antibodies to detect the N-terminal fragments of Flag-GSDMD cleaved at D87 or D275. Myc-tagged caspase-1, -4 and -5 were detected with tag specific antibodies. Bcl-2 and GAPDH were detected with specific antibodies. **d** Bcl-2 recombinant protein reduces the cleavage of GSDMD driven by active caspase-3 in vitro. The immunoprecipitated Flag-GSDMD served as a caspase-3 substrate and was incubated with active caspase-3 with or without Bcl-2 protein in caspase assay buffer. The upper panel shows GSDMD full length, merged IgG heavy chain, IgG light chain and cleaved GSDMD N-terminal P10 at longer exposure (LE). The middle panel shows the shorter exposure (SE). The bottom panel shows the added Bcl-2 recombinant protein.

It has been reported that apoptotic stimulus can induce caspase-3 or -7 mediated cleavage of GSDMD at residue D87^13^. We had detected a P10 NT GSDMD cleaved band following Bcl-2 co-expression along with the different caspases in HEK-293T cells. As the P10 NT GSDMD fragment is consistent with GSDMD D87 cleavage we sought further evidence to support this possibility. To do so we expressed Flag-GSDMD, Flag-GSDMD D275A, or Flag-GSDMD D87A in HEK 293T cells along with caspase-1, 4 or 5, in the presence or absence of Bcl-2. An antibody raised against amino acids 154–252 of GSDMD was used to detect the 88–484 C-terminal fragment of GSDMD (P42.8 CT). Following expression of caspase-1, 4 or 5, we detected the P42.8 CT cleaved bands in the samples where we had expressed Bcl-2 along with GSDMD or GSDMD D275A but it did not appear with GSDMD D87A. We reblotted the same membrane using Flag antibodies to detect the N-terminal tagged fragment of GSDMD. Again, the P10 NT bands appeared in the GSDMD and GSDMD D275A samples, but not in the GSDMD D87A sample (Fig. 3a–c). To address whether Bcl-2 also inhibits the cleavage of GSDMD at D87 by caspase-3, we performed an in vitro caspase-3 assay with immunoprecipitated GSDMD as a substrate. We found that the added Bcl-2 recombinant protein clearly impaired caspase-3 driven cleavage of GSDMD at D87 (Fig. 3d). These data indicate that the presence of Bcl-2 promoted caspase directed cleavage of GSDMD at D87 rather than at D275 in the cells, but inhibited D87 cleavage by caspase-3 in vitro.

### Manipulated expression of Bcl-2 impacts NLRP3 inflammasome activation and GSDMD cleavage

**Fig. 4:**
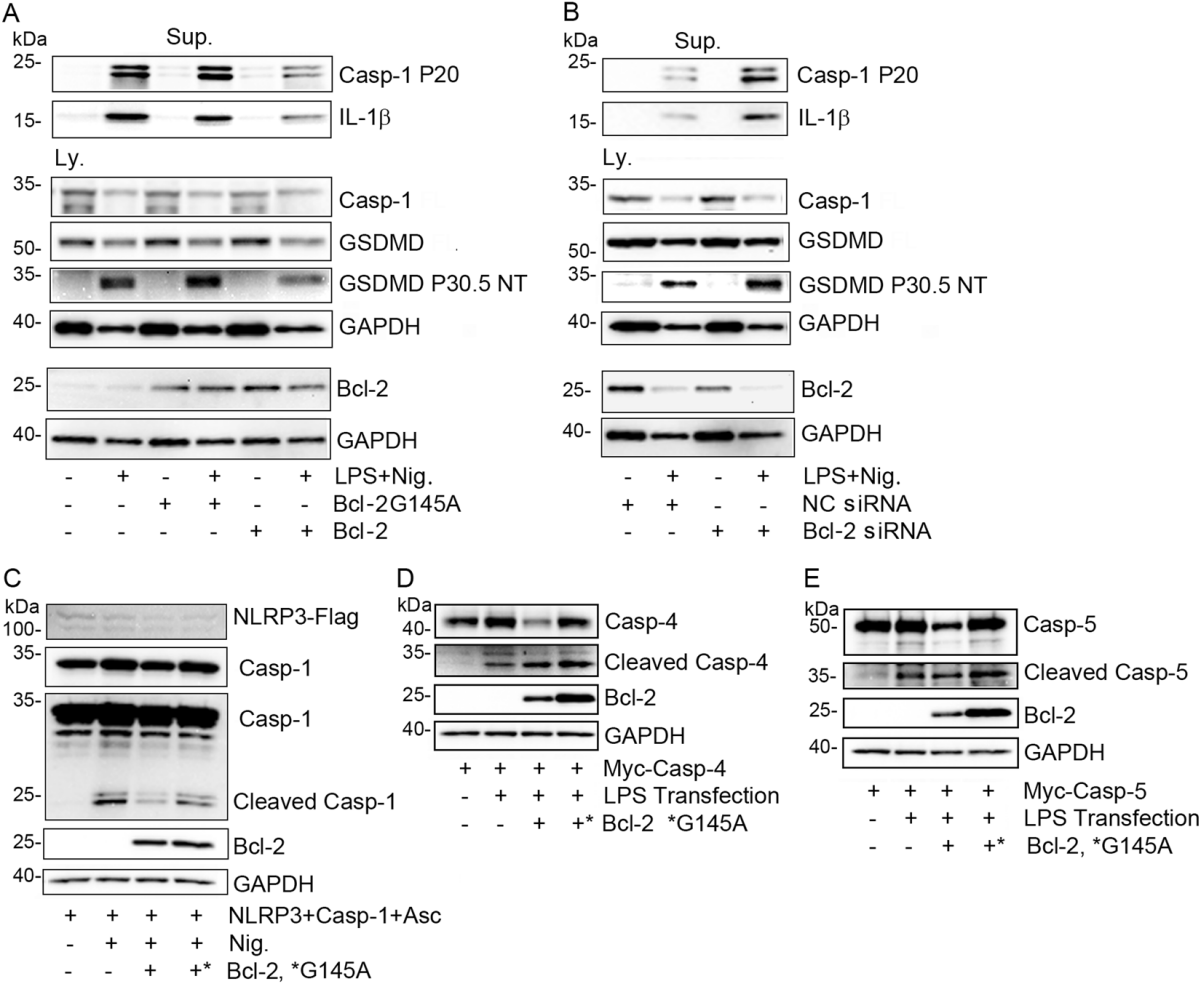
Bcl-2 expression levels in THP-1 cells affect NLRP3 inflammasome activation. **a** Expression of Bcl-2 but not Bcl-2 G145A in THP-1 cells impairs LPS and nigericin (Nig.) triggered inflammasome activation. Precipitated proteins from cell supernatants (Sup.) and from cell lysates (Ly.) were immunoblotted for the indicated proteins. **b** A siRNA directed against Bcl-2 or a scramble control siRNA were transfected into THP-1 cells. The cells were treated with LPS and nigericin and precipitated proteins from cell supernatants and the cell lysates were used for immunoblotting the indicated proteins. **c** NLRP3 inflammasome was reconstituted in HEK-293T cells by exogenous expression of all needed elements. Nigericin addition induced inflammasome activation, which was monitored by the presence of cleaved caspase-1. **d**, **e** Bcl-2 expression does not impact cytosol LPS triggered caspase-4 or -5 activation. Cell lysates prepared from HEK-293T cells transfected with the indicated constructs in the presence or absence of LPS transfection. The indicated proteins were detected by immunoblot analysis. The presence of intracellular LPS triggers caspase-4 and caspase-5 cleavage.

NLRP3 inflammasome activation leads to caspase-1 mediated cleavage of GSDMD at D275. The released N-terminus inserts into plasma membranes forming transmembrane oligomers, which assemble pores that facilitate mature IL-1β secretion^19^. To address whether Bcl-2 affected NLRP3 inflammasome induced IL-1β production, we used differentiated THP-1 cells and manipulated their Bcl-2 expression levels. Besides IL-1β secretion, we monitored caspase-1 activation and GSDMD cleavage. Following inflammasome activation, an increase in Bcl-2 levels reduced GSDMD cleavage at D275, caspase-1 activation, and IL-1 β secretion, while Bcl-2 G145A did not have these effects (Fig. 4a). Conversely, reducing Bcl2 expression prior to inflammasome activation enhanced GSDMD cleavage at D275, caspase-1 activation, and IL-1β secretion (Fig. 4b). Next, we checked caspase-1 activity following NLRP3 inflammasome reconstitution in HEK293T cells that co-expressed Bcl-2, Bcl-2 G145, or a control. Bcl-2 reduced caspase-1 activation (Fig. 4c). In contrast, when LPS trigged caspase-4 or 5 activation in reconstituted HEK 293T cells Bcl-2 expression did not meaningfully impact caspase-4 or 5 activation (Fig. 4d, e).

### Bcl-2 interacts with MLKL

**Fig. 5:**
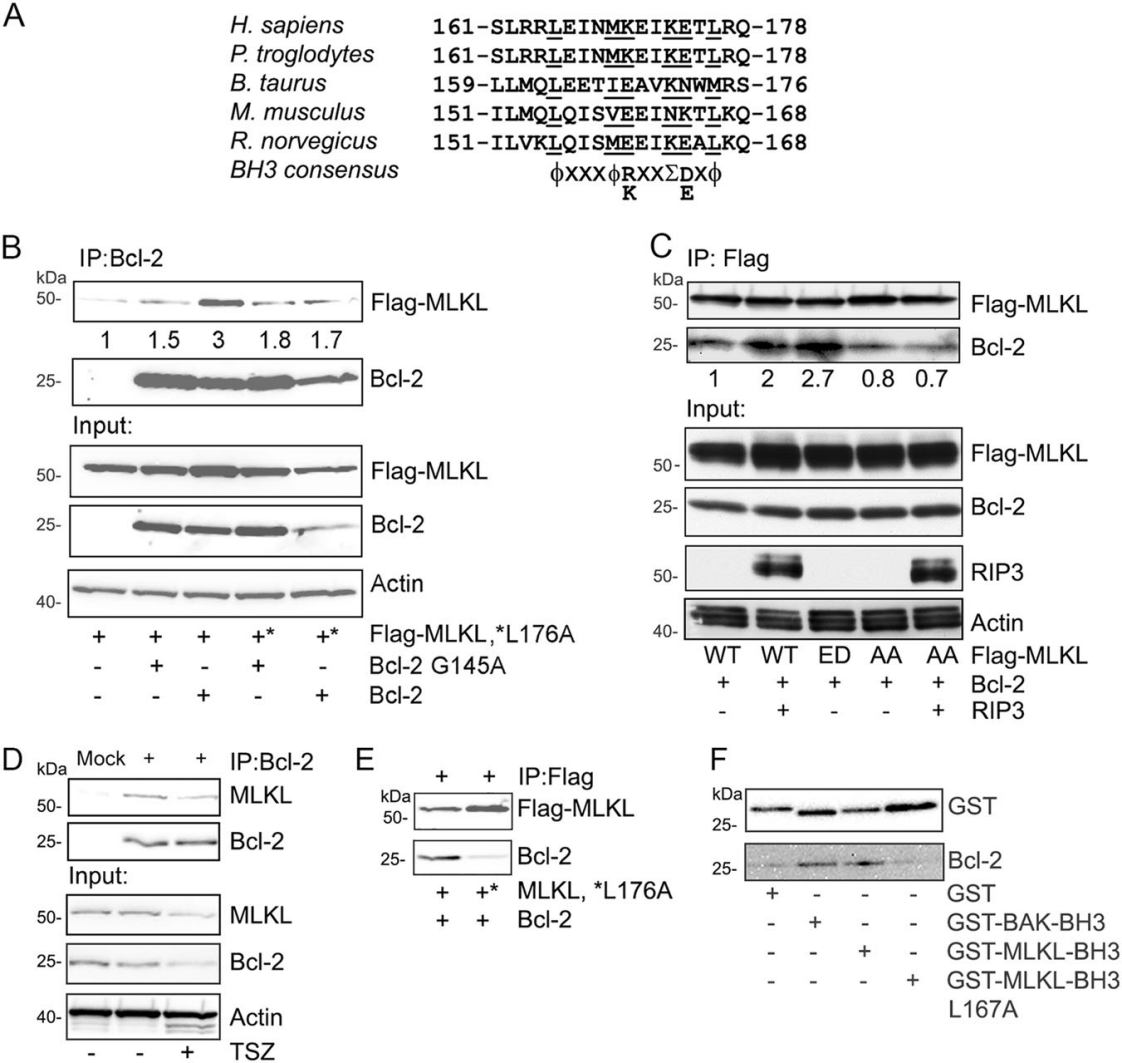
Bcl-2 interacts with human MLKL based on the presence of a BH3-like domain. **a** A predicted BH3-like domain in human MLKL is partially conserved across the indicated species. The consensus sequence for a BH3 domain is shown below. **b** Human MLKL interacts with Bcl-2. Various combinations of Flag-MLKL, Flag-MLKL L176A, Bcl-2, and Bcl-2 G145A were transfected into HEK-293T cells. Bcl-2 antibodies were used to immunoprecipitate the Bcl-2 proteins. The upper two blot panels show the co-immunoprecipitated Flag-MLKL and the immunoprecipitated Bcl-2 proteins. The lower three panels show the input proteins in cell lysates. **c** Phosphorylated MLKL interacts better with Bcl-2. Flag-MLKL, Flag-MLKL TS357-58ED (ED), and Flag-MLKL TS357-58AA (AA) were expressed in HEK-293T cells. The wild type and the AA mutant were also co-expressed with RIP3 The ED mutant is a phosphomimetic of RIP3 phosphorylated MLKL while the AA mutant cannot be phosphorylated at TS357-58. Anti-flag antibodies were used for co-immunoprecipitating Bcl-2. Western blots show all indicated proteins expressed in HEK-293T cells. **d** The endogenous Bcl-2 interacts the endogenous MLKL in THP-1 cells. The PMA treated THP-1 were used for immunoprecipitating Bcl-2 with or without treatment of TNFα 20 ng/ml (T), 100 nM Smac mimetic (S) and 20 µM z-VAD-FMK (Z) for 3 h. The lysates were used d for Bcl-2 immunoprecipitations. The pulled down of MLKL and the input proteins were blotted. (**e**) Bcl-2 recombinant protein binds with MLKL in vitro. The immunoprecipitated MLKL and its L176A mutant from HEK 293T cells were incubated with Bcl-2 protein for 1 h, and then heavily washed. Blots show the indicated proteins. **f** GST fusion BH3-like domain of MLKL binds Bcl-2 recombinant protein. The indicated GST fusion BH3 domains were expressed and purified from HEK-293T cells. Bcl-2 recombinant protein was added for in vitro binding with GST fusion BH3 domains.

The detection of a functional BH3-like domain in GSDMD prompted us to search for a similar motif in MLKL. We found a reasonable match between amino acid 165 and 176 in human MLKL. It conforms to the consensus BH3 domain sequence except at position 173, where a lysine is present while a smaller amino acid would be favored. We focused on human MLKL as the mouse sequence over the homologous region lacks a basic residue at position 160 (Fig. 5a). The purported BH3 domain in MLKL overlaps with the second brace helix (aa 169–179) in the protein. Together with the first brace helix (aa 129–158), they mediate two functional roles for the protein. First, they transmit conformational information induced by phosphorylation of the MLKL kinase domain to the N-terminal four-helix bundle (4HD) domain, and second, they provide an interface for MLKL oligomerization^20,21^.

To determine whether the identified potential BH3-like domain in human MLKL can mediate an interaction with Bcl-2, we transfected HEK 293T cells with MLKL and MLKL L176A, a mutation likely to disrupt a MLKL and Bcl-2 interface. We co-transfected Bcl-2 or its BH3 binding deficient mutant Bcl-2 G145A. We found that Bcl-2 co-immunoprecipitated with MLKL, while the MLKL L176A mutation reduced the interaction. The MLKL wild type and its L176A mutant both reduced in the Bcl-2 G145A immunoprecipitations (Fig. 5b). These suggest that the interaction depended upon Bcl-2 recognizing the BH3-like domain in MLKL.

RIP3 phosphorylates the MLKL pseudokinase domain, which switches MLKL from an inert to an activated state^16^. To test whether the phosphorylation status of MLKL impacted the MLKL/Bcl-2 interaction, we utilized a MLKL phosphorylation site deficient mutant MLKL T357A/S358A and its phosphorylation mimic MLKL T357D/S358E. Constructs expressing each of the proteins were transfected into HEK 293T cells along with RIP3 and Bcl-2. We assessed the interaction between the MLKL proteins and Bcl-2 (Fig. 5c). The results showed that either expressing RIP3 with wild type MLKL or expressing the MLKL phosphorylation mimic enhanced the interaction between MLKL and Bcl-2 (Fig. 5c). In contrast, expression of the phosphorylation site deficient MLKL protein slightly reduced the interaction, even when co-expressed with RIP3 (Fig. 5c). These data suggest that the phosphorylation status of the pseudokinase domain in MLKL can modulate the affinity of the interaction between MLKL and Bcl-2. To examine whether the endogenous MLKL interacts with Bcl-2, THP-1 cells were chosen for immunoprecipitation assays. We found that Bcl-2 consistently interacted with MLKL (Fig. 5d). Moreover, MLKL bound to Bcl-2 recombinant protein, and the BH3-like domain mutant L176A of MLKL exhibited less binding (Fig. 5e). To prove BH3-like domain of MLKL alone could bind with Bcl-2 recombinant protein, we expressed and purified GST fusions of BH3-like domain of MLKL, its mutant L176A, and the BAK BH3 domain. The protein binding assays showed that the GST-MLKL BH3-like domain bound Bcl-2, and the L176A mutation reduced its interaction with Bcl-2 (Fig. 5f). Taken together, our data indicate that the MLKL BH3-like domain can bind Bcl-2.

### Bcl-2 reduces phosphorylation of MLKL and decreases the formation of MLKL oligomers

**Fig. 6:**
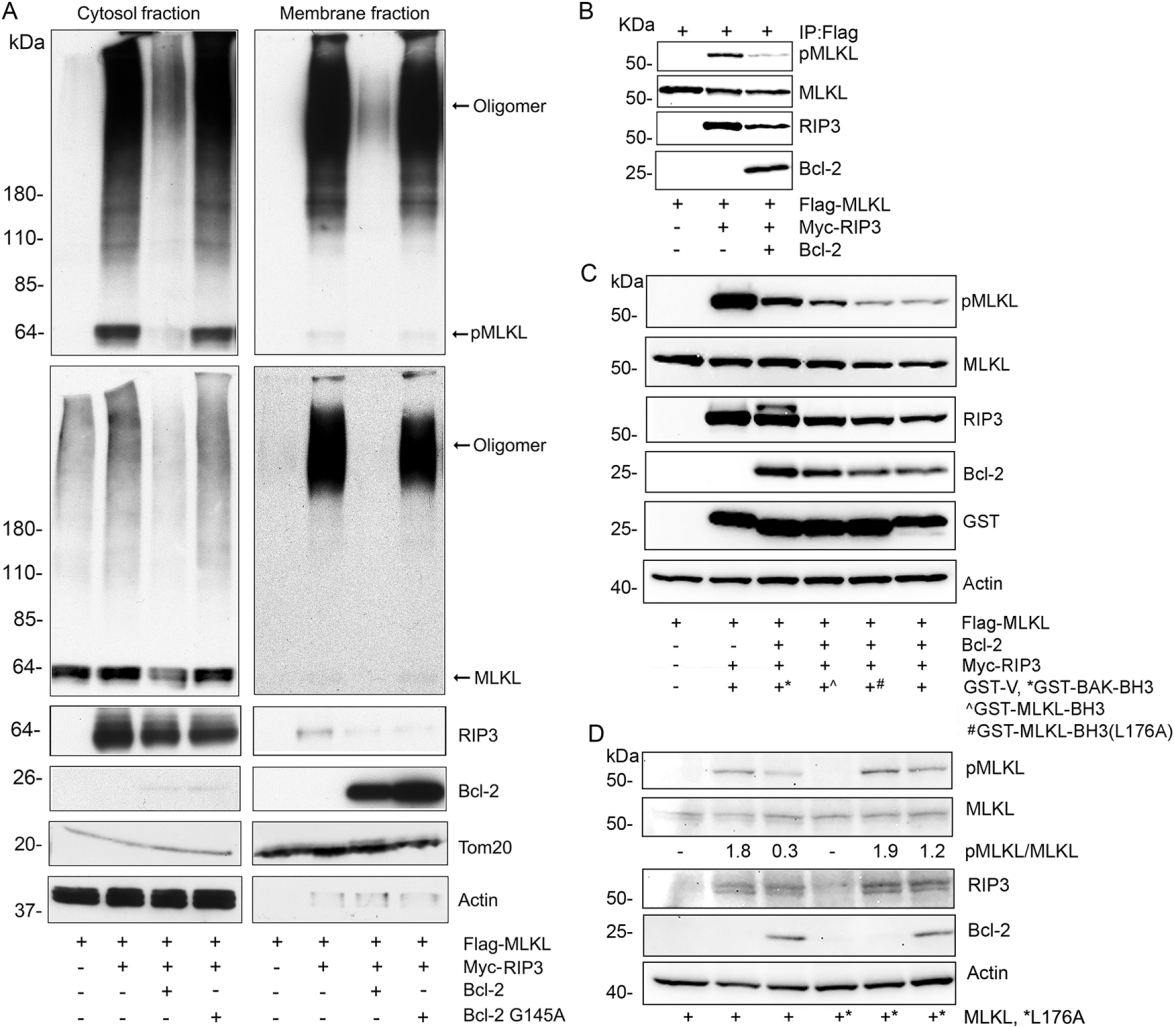
Transient expression of Bcl-2, but Bcl-2 G145A mutant impairs RIP3 induced MLKL phosphorylation and oligomerization. **a** Cell lysates from HEK-293T cells transfected with the indicated constructs were separated into cytosol and membrane enriched fractions using a detergent-based phase separation method. The indicated proteins in the two fractions were immunoblotted. The membranes were first blotted with anti-phospho-MLKL antibody (upper panel), and then reblotted with anti-Flag antibody (large middle panel). Bcl-2 and RIP3 immunoblots are shown in the lower panels. Actin and the TOM20 immunoblots verify equal loading of the cytosol and membrane fractions. **b** In vitro kinase assay. The immunoprecipitated and purified Flag-MLKL served a substrate of RIP3 kinase, which was expressed in HEK-293T cells. Phospho-MLKL (S358) antibodies were used to detect the amounts of phosphorylated MLKL. Bcl-2 recombinant protein was added to the assay. Immunoblots show all indicated proteins. **c** GST fusion BH3-like domain of MLKL reduces Bcl-2’s inhibition of RIP3 mediated MLKL phosphorylation. The indicated proteins were expressed in HEK-293T cells, the lysates were used for immunoblots. The immunoblotted proteins are shown. **d** The MLKL L176A mutation interferes with Bcl2’s inhibition of RIP3 mediated MLKL phosphorylation. The indicated proteins were transiently expressed in HEK-293T cells and identified by immunoblotting.

**Fig. 7:**
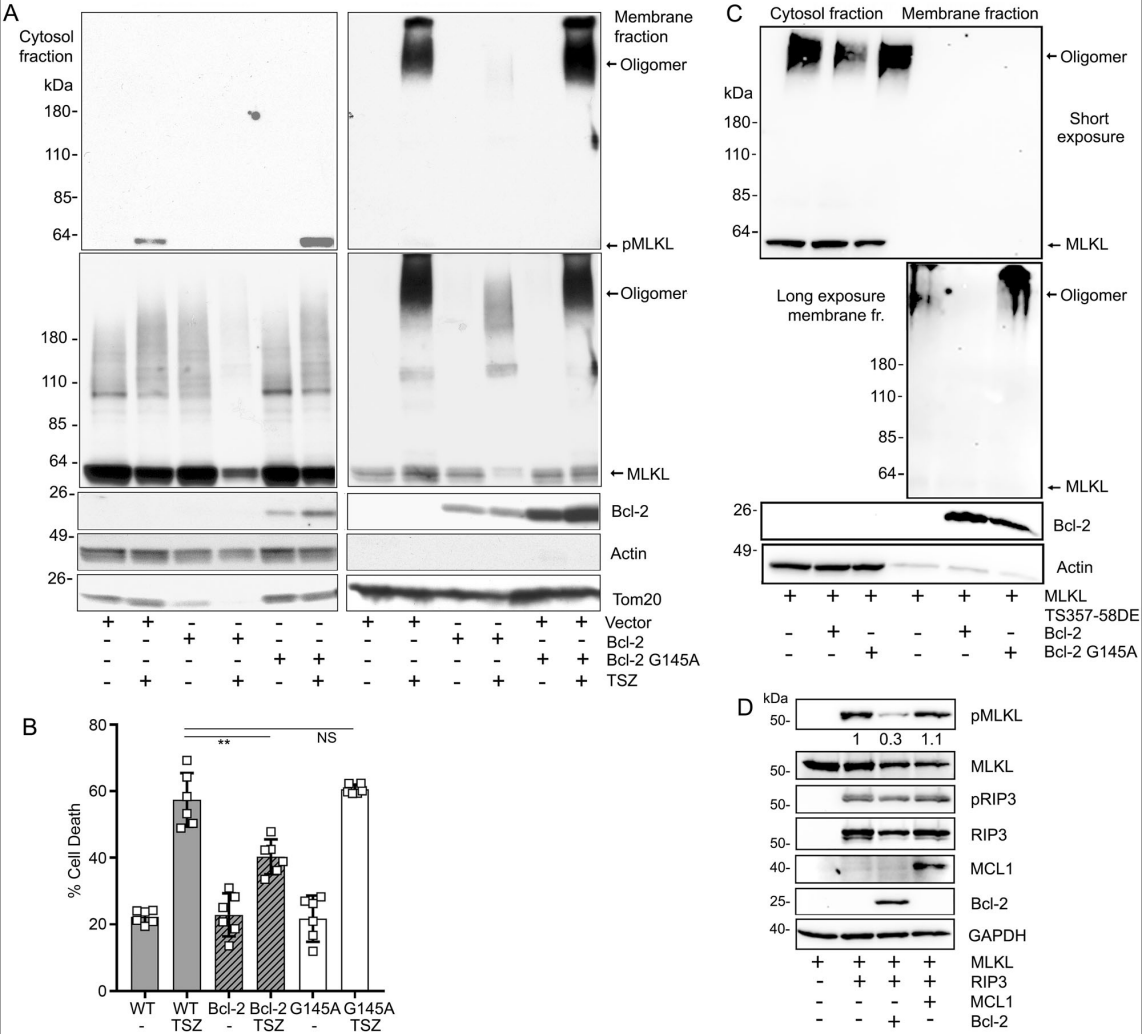
Bcl-2 regulates TSZ induced-necroptosis in HT-29 cells. **a** Stable expression of Bcl-2, but Bcl-2 G145A decreases TSZ induced-MLKL phosphorylation and oligomerization. The HT-29 cells stably expressing a vector control, Bcl-2, or Bcl-2 G145A were treated with TNF-α 20 ng/ml (T), 100 nM Smac mimetic (S) and 20 µM z-VAD-FMK (Z) for 5 h. DMSO served as a vehicle control treatment. The stimulated cells were fractionated into the cytosol and membrane fractions by detergent phase separation and subjected to immunoblotting. pMLKL (top panel), total MLKL (2nd panel). Bcl-2, Actin, TOM20 levels are shown in the lower blots. **b** Bcl-2 reduces TSZ induced cell death. Stably transfected HT-29 cells were stimulated with TSZ 12 h, and then the cells were stained with SYTOX green to assess cell death. Data are means and ± SD of four samples. **p < 0.01. **c** Bcl-2, but not Bcl-2 G145A reduces the oligomerization of MLKL phosphomimic protein. HEK-293T cells were transfected with the indicated constructs. Cell lysates were subjected to detergent phase separation. The indicated proteins are shown by western blot analysis of the cytosol and membrane enriched fractions. A longer exposure of the membrane fraction is shown below the short exposure. The Actin and TOM20 blots served as loading controls. **d** Bcl-2, but not MCL1 impairs RIP3 induced-MLKL phosphorylation. HEK-293T cells were transfected with constructs expressing MLKL, RIP3, and either MCL1 or Bcl-2. The proteins were detected by immunoblotting. The first two panels show pMLKL and total MLKL. The third and fourth panels show pRIP3 and total RIP3. MCL1 and Bcl-2 levels are on the fifth and the sixth panel. The last panel shows the loading control GAPDH.

The above results encouraged us to check the impact of Bcl-2 on MLKL functionality. First, we expressed RIP3 to drive MLKL phosphorylation and oligomerization in HEK 293T cells, which lack endogenous RIP3. We co-transfected either Bcl-2 or Bcl-2 G145A. The cell lysates were separated into membrane and cytosol enriched fractions to check the status of MLKL phosphorylation and oligomerization. We found that the wild type Bcl-2, not only reduced MLKL phosphorylation, but also its oligomerization. In contrast, Bcl-2 G145A had little impact (Fig. 6a). Second, an in vitro kinase assay using RIP3 and MLKL showed that the addition of a Bcl-2 recombinant protein reduced MLKL phosphorylation (Fig. 6b). Third, the purified GST-MLKL BH3-like domain reversed the Bcl-2 mediated interference of RIP3 mediated MILK phosphorylation. Furthermore, the GST-MLKL BH3-like domain mutant L176A had less effect (Fig. 6c). Fourth, in HEK 293T cells Bcl-2 reduced MLKL phosphorylation by RIP3, and the MLKL L176A mutation reduced the efficacy of Bcl-2 (Fig. 6d). Taken together, our results indicate that BH3-like domain of MLKL facilitates Bcl-2 mediated inhibition of MLKL phosphorylation by Rip3. Furthermore, we made use of HT-29 cells, a human colon adenocarcinoma cell line. Treating these cells with a combination of TNF-α, molecules that sensitize TNF-α activity such as second mitochondria-derived activator of caspases (Smac) mimetics, and a pan caspase inhibitor causes them to undergo necroptosis. This combination is often referred to as TSZ. We also favored HT-29 cells because they have low endogenous Bcl-2 expression. Therefore, we stably expressed Bcl-2 or Bcl-2 G145A in HT-29 cells and used these permanent cell lines to test whether Bcl-2 affects MLKL mediated necroptosis. Like the previous experiment using HEK 293T cells, Bcl-2, but not Bcl-2G145A reduced MLKL phosphorylation and oligomerization when induced with TSZ (Fig. 7a). The cellular viability assay showed that Bcl-2, but not Bcl-2 G145A reduced TSZ driven necroptosis (Fig. 7b).

The above studies indicated that the presence of Bcl-2 reduced MLKL phosphorylation and MLKL oligomerization. To try and tease out whether Bcl-2 directly impacted MLKL oligomerization, we co-transfected constructs expressing Bcl-2 or Bcl-2 G145A with MLKL T357D/S358E phosphorylated mimic protein into HEK 293 T cells. The MLKL mutations lessened its translocation to cellular membrane. Consequently, the majority of the oligomerized MLKL remained in the cytosol fraction. We found that Bcl-2, but not Bcl-2 G145A decreased the MLKL oligomerization by approximately 30% suggesting that Bcl-2 directly interferes with MLKL oligomerization (Fig. 7c). Finally, we tested whether the Bcl-2 family member MCL1 could play the same as role Bcl-2. We compared the effect of Bcl-2 to that of MCL1 on MLKL phosphorylation in HEK 293T cells transiently expressing RIP3 and MLKL. As previously Bcl-2 reduced the amount of phosphorylated MLKL (approximately 70%), however, MCL1 had little effect suggesting that MCL1 does not impact RIP3 driven necroptosis. We also checked whether Bcl-2 or MCL1 affected the autophosphorylation of RIP3 by immunoblotting for pRIP3. However, neither Bcl-2 or MCL1 had an appreciable effect (Fig. 7d). This suggests that Bcl-2 does impact MLKL phosphorylation by directly interfering with RIP3 kinase to target its substrate MLKL.

## Discussion

Necroptosis and pyroptosis depend upon oligomerized proteins inserting into cell membranes to form pores and cause cell death^22^. In this study, we found that Bcl-2 interacted with the pyroptosis and necroptosis effector proteins that oligomerize and insert into the plasma membrane, GSDMD and MLKL, respectively. The interaction of Bcl-2 with GSDMD and MLKL depended upon sequence motifs in them that resemble the consensus sequence for the BH3 domains present in Bcl-2 family members. Following necroptotic signals Bcl-2 reduced MLKL phosphorylation, sharply decreased its tendency to oligomerize, and reduced cell death.

Our results indicate that the binding of Bcl-2 to GSDMD alters the caspase 1, 4, and 5 mediated cleavage from residue D274 to D87, thereby resulting in the generation of less active GSDMD (fragment amino acids 1–275). We never detected the 88–257 amino acid fragment suggesting that Bcl-2 redirects the caspase from D274 to D87, rather than permitting cleavage at both sites.

Interestingly, in mouse models of sepsis the transgenic expression of Bcl-2 in a variety of cell types reduces cell death and improves survival^23^. Arguing that Bcl-2 may not only target apoptosis, but also pyroptosis, GSDMD is a likely contributor to the pathogenesis of sepsis^24^. Furthermore, a recently developed chemical inhibitor of gasdermin D, Necrosulfonamide improves survival following sepsis in mice arguing that gasdermin D inhibitors may be efficacious in treating certain inflammatory diseases^25^. Based on our study, enhancing Bcl-2 activity, which would limit GSDMD activation could also prove beneficial.

Bcl-2 has been reported to negatively affect NLRP3 inflammasome activity in macrophages^26^. Our data indicate that Bcl-2 directly inhibits caspase 1 mediated cleavage of GSDMD at D274, which will reduce the incidence of pyroptosis and lessens IL-1β release. Bcl-2 and Bcl-XL also suppress NLRP1 inflammasome activation by reducing the binding of ATP to NLRP1, suppressing NLRP1 oligomerization, and thereby reducing NLRP1-mediated caspase-1 activation. When exposed to a NLRP1 activator, Bcl-2-deficient macrophages exhibit more caspase-1 processing and IL-1β production, whereas Bcl-2-overexpressing macrophages have the opposite phenotype^27,28^. Since NLRP1 inflammasomes can also trigger pyroptosis, Bcl-2 may also directly suppress pyroptosis by and IL-1β secretion by directly altering the cleavage of GSDMD.

It has been reported that MLKL adopts a monomeric conformation in human cells prior to exposure to a necroptotic stimulus^29^. Based on our data, Bcl-2 may interact with monomeric MLKL, and with the monomeric MLKL/Rip3 complex. The presence of a BH3-like domain in MLKL that overlies the second brace region argues that the Bcl-2/MLKL interaction has a physiologic role. In the present study, Bcl-2 interactions with this region in MLKL disrupted the phosphorylation and oligomerization of the protein. Furthermore, mutation of the fourth hydrophobic residue in the human MLKL BH3-like domain (L176A) disrupted the interaction of Bcl-2 with MLKL. A mutation in mouse MLKL also in the fourth hydrophobic residue of the BH3-like domain (L166A) along with an adjacent mutation (T165A) caused defective MLKL oligomerization and interfered with the execution of necroptosis^20^.

That Bcl-2 binds to a BH3-like domain in a non-Bcl2 family member to affect function of that protein is not unprecedented. Bcl-2 binds to a BH3- like domain in Beclin-1, which acts as a brake on the initialization of autophagy^30^. Mice carrying a Beclin-1 F121A mutation, which disrupts the Belcin-1/BCL-2 autophagy regulatory complex, have increased autophagy, a prolonged duration of good health, and enhanced longevity^31^. Our investigation provides two other examples of Bcl-2 acting on non-Bcl-2 family to regulate protein function.

## Materials and methods

### Reagents and antibodies

Antibodies used for immunoblotting analysis were the following: anti-IL-1β (#12703), anti-caspase-1 (#3866), anti-caspase-4 (#4450), anti-caspase-5 (#46680), anti-MLKL (#14993), anti-phospho-MLKL (#91689), anti-RIP-3 (#13526), anti-phospho-RIP3 (#93654) and anti-Myc-tag (#2278) (Cell Signaling Technology); and anti-Flag (Sigma-Aldrich, F1804), anti-Bcl-2 (Santa Cruz Biotechnology, sc-783), anti-actin conjugated to horseradish peroxidase (Sigma-Aldrich, A3854), anti-GAPDH conjugated to horseradish peroxidase (Proteintech, HRP-60004), goat anti-rabbit HRP-linked antibody (#7074), and horse anti-mouse HRP-linked antibody (#7076) (Cell Signaling Technology). Anti-GSDMD was from (Novusbio, NBP2-33422). The LPS, nigericin and Z-VAD-FMK caspase inhibitor were purchased from Sigma-Aldrich. SYTOX Green nucleic acid stain was purchased from Invitrogen. Smac mimetic was from Tocris. Caspase-1, caspase-3 and Bcl-2 recombinant proteins were from EMD Millipore. Human TNFα was from R&D System. Smac mimic was from APExBIO.

### Cells, plasmids, and siRNAs

THP-1 cells were obtained from the American Type Culture Collection. THP-1 and HT-29 cells were maintained in RPMI 1640 Medium supplemented with 10% FBS (Invitrogen), and HEK 293 T cells were maintained in DMEM Medium with 10% FBS. To differentiate THP-1 cells into macrophages the cells were treated with 25 ng/ml of PMA (Sigma-Aldrich) for 3 h. Subsequently, the cells were washed with Opti-MEM medium (Life Technologies) and re-seeded into 12 well plates in 0.5 ml of Opi-MEM media. The Flag tagged GSDMD full length, its NT, CT, and D275A mutant plasmids; and Myc-caspase-1, Myc-caspase-4, Myc-caspase-5 and Myc-caspase-11 were a kind gift from Dr. Feng Shao (National Institute of Biological Sciences, Beijing, Collaborative Innovation Center for Cancer Medicine, Beijing China). Flag-MLKL and Myc-RIP3 plasmids were a gift from Dr. Zheng-Gang Liu (National Cancer Institute, National Institutes of Health). GST fusion BAK BH3 (70–87 aa), GST fusion GSDMD BH3-like (142–159 aa), its mutant L150Q, GST fusion MLKL BH3-like (161–178 aa) were made by ligating the respectively synthesized DNA with a GST expression vector. Bcl-2 and scrambled control siRNAs were purchased from Santa Cruz Biotechnology. The plasmids or the siRNAs were transiently transfected into the cells using X-tremeGENE-HP (Roche) following the manufacture’s protocol.

### Stable expression of Bcl-2 and its mutant in HT-29 cells

Bcl-2 plasmids were transfected into HT-29 cells. 24 h later G418 (400 µg/ml) was added to the cell culture for 2 weeks. Dead cells were removed when the media was changed. Single clones were picked and expanded for evaluation. Bcl-2 expression was tested by immunoblotting and positive cell lines were expanded for cell freezing and experiments.

### Immunoblot analysis and immunoprecipitations

For standard immunoblotting, the cells were lysed using a buffer of 20 mM HEPES, pH 7.4, 50 mM β-glycerophosphate, 1 mM Na_3_VO_4_, 0.5% (vol/vol) Triton X-100, 0.5% (vol/vol) CHAPS (3-[(3-cholamidopropyl)-dimethylammonio]-1-propane sulfonate hydrate) and 10% (vol/vol) glycerol with a protease inhibitor ‘cocktail' tablet (Roche). The lysates were separated by SDS-PAGE and transferred to nitrocellulose membrane by iBLOT Gel Transfer System (Invitrogen). The membrane was incubated with 5% nonfat milk w/v in TBS buffer (25 mM Tris-HCl, pH 7.5; 150 mM NaCl; 0.1% Tween-20) for 1 h, and then reacted with the primary antibody in TBS buffer with 2.5% nonfat milk or 5% BSA w/v overnight by shaking at 4 °C. The appropriate second antibodies conjugated to HRP were used to detect the protein of interest via enhanced chemiluminescence (ECL). To immunoprecipitate endogenous or tagged proteins, the cells were lysed in the above buffer and incubated for 2 h at 4 °C with the antibody conjugated beads or primary antibodies followed by protein G beads for another 1 h. The captured immunoprecipitates were washed eight times with lysis buffer, then separated by SDS-PAGE, and analyzed by immunoblotting. The results were quantitated using ImageJ.

### Phase separation fractionation

The fractionation was performed as previously described^16^. Briefly, cell pellets were re‐suspended in 5× volume of Triton X-114 lysis buffer (20 mM HEPES, pH 7.4, 150 mM NaCl, 2% Triton X-114, and complete protease inhibitor [Roche]) and incubated on ice for 30 min. The cell lysate was centrifuged at 15,000 × *g* at 4 °C for 10 min, and then the supernatant was harvested as the detergent soluble fraction. After warming at 30 °C for 3 min, the detergent soluble fraction was centrifuged at 1500 × *g* for 5 min at room temperature. The aqueous layer was collected then re‐centrifuged at 1500 × *g* for 5 min to remove any contamination from the detergent enriched layer and saved as the aqueous faction. The detergent enriched layer was diluted with 20 mM HEPES, pH 7.4, 150 mM NaCl to the same volume as the detergent soluble fraction and re‐centrifuged at 1500 × *g* for 5 min. The washed, detergent enriched layer was diluted with the same buffer to the identical volume as the aqueous faction and saved as the detergent fraction.

### In vitro protein binding, protein kinase, and caspase assay

For in vitro protein binding assay, the immunoprecipitated GSDMD, MLKL or GST-BH3-like domains were washed 4 times with lysis buffer, then incubated with added Bcl-2 recombinant protein, or BSA as a control protein. The samples were washed again with lysis buffer prior to SDS-PAGE gel and immunoblotting. For in vitro kinase assays, cell lysate from HEK 293 T expressing RIP3 were added to the immunoprecipitated and purified MLKL using a kinase reaction buffer (25 mM HEPES, pH 7.4, β-glycerol phosphate 12.5 mM, MgCl_2_ 10 mM, fresh ATP 100 µM and DTT 5 mM). The sample was incubated at room temperature with gently shaking for 30 min. SDS loading buffer was added to stop the reaction prior to fractionation on SDS-PAGE gel. Similarly, in vitro caspase activation assays were performed by mixing the active caspase, Bcl-2 recombinant protein, along with immunoprecipitated and purified protein together in caspase reaction buffer (50 mM HEPES, pH 7.4, NaCl 50 mM, 0.1% CHAPS, 1 mM EDTA, 5% glycerol, fresh 10 mM DTT), for a 30 min incubation at room temperature. SDS loading buffer was added to stop the reaction prior to fractionation on SDS-PAGE gel.

### Cell viability assay

To detect cell death triggered by cleaved GSDMD, we used trypan blue uptake and light microscopy. Twenty-four hours following transfection, trypan blue stained cells were evaluated using bright light microscopy. Blue stained cells with ruptured plasma membranes were distinguished from non-stained live cells with intact plasma membrane. The dead cells were calculated as a percentage of the total number of cells^32^. SYTOX green was used to evaluate cellular necroptosis induced by TNF-α 20 ng/ml, 100 nM Smac mimetic and 20 µM z-VAD-FMK following the manufacturer’s protocol.

### Inflammasome activation assay

To assess NLRP3 inflammasome activation THP-1 cells were treated with PMA 25 ng/ml for 3 h and the cells washed once with Opi-MEM medium (Life Technologies). The cells were reseeded with 0.5 ml Opi-MEM medium in 12 well plate. LPS (50 ng/ml) was used to prime the cells overnight, and nigericin (15 µM) was added for 45 min, then the cell supernatants were collected. Bcl-2 or its siRNA were transfected into PMA treated THP-1 cells overnight. Following the culture period, the supernatants were transferred to a microcentrifuge tube and 0.5 ml of methanol and 0.125 ml chloroform added. After mixing and a 5-min centrifugation at 13,000 RPM, the upper phase was discarded being careful not to disturb the interface. 0.5 ml methanol was added the samples spun again for 5 min at 13,000 rpm. The supernatants were discarded, and the pelleted proteins air dried for 5 min at 50 °C. After which 60 µl of 1× sample loading buffer with DTT (final concentration of 0.1 µM) was added to each sample prior to SDS-PAGE and immunoblotting to detect IL-1β and caspase-1 p20.

### Statistics

All experiments were repeated a minimum of three times unless otherwise indicated. Statistical significance is based on the analysis of at least triplicate samples. Standard errors of the mean (SEM) and *p*-values were calculated using *t*-test in GraphPad Prism (GraphPad software).
